# Assessing the nature of asthma in African epidemiological studies: a scoping review protocol

**DOI:** 10.1186/s13643-020-01491-7

**Published:** 2020-10-07

**Authors:** Vuyelwa Ndlovu, Moses John Chimbari, Elopy Sibanda

**Affiliations:** 1 School of Nursing and Public Health, College of Health Sciences, Howard College Campus, University of KwaZulu-Natal, Durban, South Africa; 2 Department of Environmental Science and Health, Faculty of Applied Sciences, National University of Science and Technology, Corner Gwanda Road and Cecil Avenue, PO Box AC 939, Ascot, Bulawayo, Zimbabwe; 3 Asthma, Allergy and Immune Dysfunction Clinic, Twin Palms Medical Centre, 113 Kwame Nkrumah Avenue, Harare, Zimbabwe; 4 Department of Pathology, Medical School, National University of Science and Technology, Bulawayo, Zimbabwe

**Keywords:** Asthma, Africa, Epidemiology, Environmental exposure, Scoping review

## Abstract

**Background:**

Asthma is one of the most common chronic respiratory conditions in the world and is increasing in prevalence, particularly in Africa and other low-income countries. The disproportionately high numbers of premature deaths and severe or uncontrolled cases in many African countries are indicative of their inability to cope with a costly disease like asthma. Progress has, however, been made in understanding the complex and heterogeneous nature of the disease. The objective of this study will be to summarise the epidemiological literature on the nature of asthma in African countries.

**Methods:**

We registered a study protocol for a scoping review. The review was designed following the Arksey and O’Malley framework. We will search PubMed/MEDLINE, African Journals Online (AJOL) and relevant grey literature (e.g. Google Scholar, EBSCOhost) from January 1990 onwards. Only primary epidemiological studies of asthma (e.g. frequency, disease mechanisms, associated risk factors and comorbidities) written in English and conducted in Africa will be included. Two reviewers will independently screen all citations, full-text articles and abstract data. Potential conflicts will be resolved through discussion. Findings will be reported using narrative synthesis and tabulation of the summaries.

**Discussion:**

This scoping review will capture the state of the current epidemiological literature on asthma in African countries. Results will be published in a peer-reviewed journal. We anticipate this review will identify gaps and make recommendations for future areas of study.

**Scoping review registration:**

Open Science Framework http://osf.io/n2p87/

## Background

Asthma has become a serious public health concern [1] as the global prevalence and mortality continues to increase, particularly in African countries [2–4]. The Global Burden of Disease collaboration estimated that at least 400,000 people died prematurely from asthma, mainly in Africa and other low- and middle-income countries (LMICs) in 2016 [5]. It is a complex disease defined as a chronic inflammatory disorder of the airways associated with airway hyperresponsiveness and airflow obstruction that can be reversed either spontaneously or with treatment [3]. The symptoms include wheeze, shortness of breath, chest tightness and cough that vary over time and in intensity [3]. All age groups are affected, especially children who endure the greatest burden [2, 6]. In 2018, 339 million individuals were estimated to have asthma worldwide [7] with the number expected to exceed 400 million by 2025 if the current rising trends continue [1, 4]. The high asthma burden is characterised by poor quality of life and associated direct and indirect costs such as expensive medicine, frequent hospital admissions and absences from school or work [7–9].

Asthma is a heterogeneous disease believed to occur as a result of a complex interaction between genetic, immunological and environmental factors [10, 11]. The paradigm of asthma heterogeneity has been evolving for the past several decades with different classification methods proposed and the quest continues as we approach the era of biologics and precision medicine [12–14]. Several phenotypes have been identified to better characterise the clinical presentation, triggers and treatment response [15]. The phenotype of allergic asthma which is T-helper cell type 2 (Th2) mediated is one of the most well-understood types [16]. It usually starts in childhood, often accompanied or preceded by allergic rhinitis and/or atopic eczema and responds well to treatment. Another Th2-related phenotype is allergic bronchopulmonary mycoses (ABPM), a severe type of asthma caused by a hypersensitivity reaction to fungi typically by *Aspergillus fumigatus* [17]*.* This phenotype is reported to be increasingly common in Africa [18]. The non-allergic eosinophilic asthma phenotype which is also Th2 related often develops later in life and is often more severe than allergic asthma [19, 20]. It may occur as a result of occupational irritant exposures such as pesticides or particulate matter and the asthma symptoms are quite severe compared to childhood-onset asthma. Another adult-onset phenotype of asthma which is severe but not very well understood is neutrophilic asthma [13]. Though asthma phenotypes have been developed to be distinguishable from each other [21–23], there is some overlap which has prompted research focus into identifying the different underlying pathological mechanisms or endotypes [12]. Furthermore, genetic, environmental and lifestyle factors and other comorbidities are recognised to influence the type and stability of phenotypes over time. The proposed systems biology approach promises to address this complex interaction in a holistic manner in an effort to improve the definition of phenotypes [24, 25].

The sequential lifestyle changes that are occurring in the African continent because of rapid urbanisation [3, 5, 6] and increased exposure to environmental and occupational factors [26, 27] are believed to be accelerating the development of asthma [28]. Many African countries already have fragile health systems that are overburdened by infectious diseases [29]. The high numbers of premature deaths and severe or uncontrolled cases are evidence of their inability to cope with and accommodate a disease like asthma [30–35]. Moreover, it was reported in a survey conducted in 2013–2014 by the Global Asthma Network (GAN) that there was no national asthma strategy for both children and adults in many African countries implying the likely absence of health promotion programmes to raise community awareness about asthma and associated risk factors [36].

In light of the above evidence, the primary challenge, therefore, is to identify modifiable risk factors that can inform the design of appropriate public health interventions in order to reduce the morbidity and severity of asthma in African settings. This is not an easy task mainly because of limited specialists in the field of asthma and allergy and low locally relevant research activity [28]. Research in the field of asthma and allergy is currently concentrated in developed high-income countries (HICs) [37–39]. It is difficult to generalise the findings to African countries due to systematic differences in the genetic, environmental and lifestyle characteristics [37, 40–42]. For instance, in Africa, there is uncontrolled or unregulated exposure to many irritant pollutants such as dust, smoke and pesticides [39, 43]. The risk and intensity of exposure to these harmful exposures amongst different vulnerable population groups continues to increase due to the lack of clear regulatory policies particularly in informal occupational settings [44, 45]. Consequently, there is increasing concern about the scarcity of published asthma epidemiological studies in Africa [46]. This must be addressed in order to better understand the disease patterns and identify risk factors that predict asthma [39, 47, 48].

To facilitate progress in future epidemiological research in Africa, a clear understanding and application of current knowledge about asthma is required in order to inform the design of more robust studies. Epidemiology studies for asthma have been marred by the lack of consensus in the use of operational definitions of the disease due, in part, to the underlying heterogeneity [49]. The focus should be to address the heterogeneous nature of the disease through the characterisation of phenotypes and associated comorbidities, lifestyle or environmental factors. The utilisation of objective testing tools aids in the diagnosis and identification of various asthma phenotypes [50]. Their use should be encouraged even in low-income countries to minimise misdiagnosis in clinical settings or misclassification in epidemiological studies.

There is an urgent need to assess the degree to which asthma epidemiological research in Africa has considered asthma heterogeneity as it has a bearing on future prevention and management strategies. To achieve this, a scoping review has been preferred because of its usefulness in examining the nature and extent of research activity, mapping key concepts in the methodologies and evidence gathered and identifying gaps in the literature to inform future research [51, 52]. It is important, through this scoping review, to identify and map asthma phenotypes and associated comorbidities and environmental or lifestyle risk factors to understand how they may vary amongst African countries.

### Aim

The objective of this study will be to summarise the epidemiological literature on the nature of asthma in African countries.

## Methods

The review protocol has been registered within the Open Science Framework database (registration ID: http://osf.io/n2p87/) and is being reported in accordance with the reporting guidance provided in the Preferred Reporting Items for Systematic Reviews and Meta-Analyses Protocols (PRISMA-P) statement [53] (see checklist in Additional file 1). The proposed scoping review will be reported in accordance with the reporting guidance provided in the Preferred Reporting Items for Systematic Reviews and Meta-analyses (PRISMA) extension for Scoping Reviews (PRISMA-ScR) [54]. This scoping review was designed following the Arksey and O’Malley framework [52] that has been further refined by other authors [51, 55, 56]. A scoping review is designed to examine the extent, range and nature of research activity in a topic of interest and identify research gaps in the existing literature. The framework details six different stages in the process of conducting a good scoping review: (1) identifying the research question, (2) identifying relevant studies, (3) selecting the studies, (4) charting the data and (5) reporting the results and the optional sixth stage involving consultations with relevant stakeholders [52]. We will not have formal consultation of stakeholders in the proposed scoping review.

### Stage 1: Defining the research questions

How have asthma epidemiological studies in African settings considered the evolving disease paradigm of asthma heterogeneity?

The specific research questions that will be addressed are:

1. What are the most commonly identified asthma phenotypes in African settings?
2. To what extent have the studies considered other comorbidities and environmental and lifestyle factors?

#### Eligibility criteria

The PCC framework (‘Population–Concept–Context’) [57] was used to clearly define the concepts in the main review question.

#### Population

We will include studies involving children, adolescents and adult population (regardless of age or sex).

#### Concept

Articles reporting original research on asthma using epidemiological study designs (such as randomised trials, cohort studies, case-control, cross-sectional and case series) will be included. This includes studies with an objective measurement, diagnosis and clear operational definition of asthma, the different disease mechanisms or phenotypes, the associated risk factors and comorbidities. Animal studies, case studies and literature reviews will be excluded.

#### Context

Articles from African countries will be considered for inclusion in this review. For example, the following countries will be eligible: Algeria, Angola, Benin, Botswana, Burkina Faso, Burundi, Cabo Verde, Cameroon, Central African Republic, Chad, Comoros, Democratic Republic of the Congo, Republic of the Congo, Cote d’Ivoire, Djibouti, Egypt, Equatorial Guinea, Eritrea, Eswatini, Swaziland, Ethiopia, Gabon, Gambia, Ghana, Guinea, Guinea-Bissau, Kenya, Lesotho, Liberia, Libya, Madagascar, Malawi, Mali, Mauritania, Mauritius, Morocco, Mozambique, Namibia, Niger, Nigeria, Rwanda, Sao Tome and Principe, Senegal, Seychelles, Sierra Leone, Somalia, South Africa, South Sudan, Sudan, Tanzania, Togo, Tunisia, Uganda, Zambia and Zimbabwe. Studies conducted outside the African continent will not be considered, even if the population under study includes those of African descent.

Articles in English published since January 1990 onwards will be selected as this will capture the period in the evolution of asthma heterogeneity, including when there was a resurgence of interest in defining asthma subsets [12].

### Stage 2: Identifying relevant studies

The primary source of literature will be a structured search of several electronic databases (from January 1990 onwards): PubMed/MEDLINE, African Journals Online (AJOL), African Index Medicus (AIM), Global Health and EBSCOhost. The secondary source of potentially relevant material will be a search of the grey or difficult to locate literature, including OpenGrey, Open Access Theses and Dissertations (OATD), Google scholar, the websites of the World Health Organization (WHO) and other organisations. We will perform hand-searching of the reference lists of included studies, relevant reviews or other relevant documents. Content experts and authors who are prolific in the field will be contacted. The literature searches will be designed and conducted by the review team. A librarian will be consulted to assist in developing a final search strategy. The search strategy will be customised to each selected database, and the adaptations will be reported in the planned review. The search will include a broad range of terms and keywords related to asthma, epidemiological studies and the geographical area ‘Africa’. A draft search strategy for PubMed/MEDLINE is provided in Additional file 2.

### Stage 3: Study selection process

All the citations from the selected databases, the grey literature and other sources will be imported into EndNote X8 (Clarivate Analytics, PA, USA). Before the screening of citations begins, all duplicate citations will be removed manually. The screening process will be in two steps. The first step will involve the first author (VN) and a second reviewer each independently screening the title and abstract of all retrieved citations for eligibility based on the specified inclusion and exclusion criteria. This will then be reviewed by the rest of the research team and, on consensus, move to the next step where relevant citations will be included in the full-text review. The first author (VN) and a second reviewer will independently review the full texts to assess eligibility using the specified criteria. The rest of the research team will again review this process until full consensus is attained. If disagreements arise between VN and a second reviewer at both stages of the screening process, a third reviewer (MC) will be brought in as a moderator and make the final decision.

### Stage 4: Extracting and charting the data

A data charting form will be developed that addresses the research questions and the aim of the scoping review. Since the focus of the proposed review is on epidemiological studies, it was fitting for the data charting form’s design to be informed, to some extent, by the Strengthening the Reporting of Observational Studies in Epidemiology (STROBE) checklist [58]. The STROBE checklist was developed to provide general reporting recommendations for descriptive observational studies such as cohort, case-control and cross-sectional studies [58]. Therefore, the Joanna Briggs Institute Reviewer’s data extraction instrument [57] will be modified to incorporate some aspects of the STROBE checklist in order to extract all relevant information relating to the population, concept and context. The current template of the data charting form is presented in Additional file 3.

It is anticipated that during the review, this will be an iterative process whereby the data charting form will be continually updated as the research team becomes more familiar with the literature and important themes are identified. The data extracted will be discussed by the research team then summarised and tabulated in themes that address the research questions. Data to be extracted will include, but not be limited to, publication year, study design, country, study setting, population characteristics, sampling and recruitment of participants, data collection, exposure and outcome variables of interest and their operationalization, characteristics of study participants, identified environmental risk factors, confounders and important conclusions reached from the study.

### Stage 5: Collating, summarising and reporting the results

The study selection process will be summarised in a flow chart adapted from the Preferred Reporting Items for Systematic Reviews and Meta-Analyses (PRISMA) Statement [59]. The data from each paper (e.g. study characteristics, context, participants, outcomes, findings, limitations) will be used to build evidence tables of an overall description of included studies.

The data arising from our data collection process will be summarised quantitatively (using a simple numerical count) and qualitatively (drawing on the descriptive analytical method) using thematic analysis and visual representations (including maps or diagrams). Through this process, we will be able to identify where gaps exist in the literature, as well as the research area(s) which require a systematic review or primary research. We will use our results to (1) determine the most commonly studied asthma phenotypes in African settings and (2) the extent to which asthma epidemiological studies have considered comorbidities and environmental and lifestyle factors.

## Discussion

Countries that successfully developed and implemented asthma strategies, as in the case of Finland, have seen a reduction in disease burden [36, 60]. The success of these national asthma strategies has mainly been attributed to political will, evidence-based policy making and health care, funding and capacity building [36, 60]. Extending this approach to African countries could have the same positive effect on asthma burden. This scoping review will, therefore, generate an evidence map of the available epidemiological literature on the heterogeneous nature of asthma in Africa where potential knowledge gaps will be identified. Epidemiological studies were selected as the focus of this review because they offer the best methodologies for measuring the distribution of asthma as well as identification of a wide range of risk factors explaining disease risk and severity in a population [61]. Furthermore, the use of a well-known scoping review methodology and the Preferred Reporting Items for Systematic reviews and Meta-Analyses extension for Scoping Reviews (PRISMA-ScR) guidelines [54] will enhance the quality of the results and conclusions drawn. We anticipate that the findings from this review will contribute towards the current body of knowledge and trigger dialogue on key issues for asthma research amongst interested parties. This will improve the rigour of future epidemiological studies conducted in Africa and the production of much needed evidence for the design of effective interventions.

Due to the lack of resources required for reviewing non-English literature, we recognise the impact this limitation will have on our findings. Africa is the most linguistically diverse continent in the world and English is not the only official language [62]. If there is little to no representation of eligible studies from the non-English speaking countries, a review for those specific geographical areas will be strongly recommended. Selection bias may also be introduced by the exclusion of studies published before 1990. The cut-off period, however, was selected because it is around the time when there was a resurgence of interest in defining asthma subsets [12]. Despite incorporating a grey literature search, it is probable that not all relevant studies will be identified. Furthermore, there will be no quality assessment of the studies selected as that is beyond the scope of this type of review.

Ethical approval is not required as only previously published and publicly available material will be utilised for this scoping review. Any amendments made to this protocol when conducting the study will be outlined and reported in the final manuscript. We will disseminate the findings of this scoping review through publication in a peer-reviewed journal and presentation at relevant conferences and through interaction with local stakeholders in asthma research, policy making and clinical practice.

## Supplementary information

**Additional file 1.** PRISMA-P 2015 Checklist.

**Additional file 2.** Draft search strategy from PubMed/MEDLINE.

**Additional file 3.** Data charting form.

## Data Availability

All data generated or analysed during this study will be included in the published scoping review article and will be available upon request.

## Abbreviations

PRISMA-ScR: Preferred Reporting Items for Systematic reviews and Meta-Analyses extension for Scoping Reviews checklist
AJOL: African Journals Online
HICs: High-income countries
LMICs: Low- and middle-income countries
Th2: T-helper cell type 2
ABPM: Allergic bronchopulmonary mycoses
ISAAC: International Study of Asthma and Allergies in Childhood
ECRHS: European Community Respiratory Health Survey
GAN: Global Asthma Network
PCC: ‘Population–Concept–Context’ framework
AIM: African Index Medicus
MeSH: Medical Subject Headings
OATD: Open Access Theses and Dissertations
WHO: World Health Organization
STROBE: Strengthening the Reporting of Observational Studies in Epidemiology checklist
FeNO: Fractional exhaled nitric oxide
