# Supplementary material for: Assessing the nature of asthma in African epidemiological studies: a scoping review protocol
Source: Syst Rev. 2020 Oct 7;9:230. doi: 10.1186/s13643-020-01491-7 (PMC7539529; doi:10.1186/s13643-020-01491-7)
Supplement: Supplementary file 2 — Additional file 2. Draft search strategy from PubMed/MEDLINE. [file 13643_2020_1491_MOESM2_ESM.docx]

**Additional File 2: Summary of preliminary search strategy**

| **PCC element** | **Search strategy** | **Strategy Explanation** |
| --- | --- | --- |
| **Population (P)** | 1. Adult [MeSH Terms] 2. Adolescent [MeSH Terms] 3. Child[MeSH Terms] 4. Search (((((Male[Text Word]) OR Female[Text Word]) OR Children[Text Word]) OR Adult *[Text Word]) OR Worker *[Text Word]) OR Adolescen*[Text Word] 5. 1 OR 2 OR 3 OR 4 | Search sets 1-5 are the**MeSH terms** and**text words** for the **population**. They are combined using the Boolean term **OR.** |
| **Concept (C)** | 1. (((((Epidemiology[MeSH Terms]) OR etiology[MeSH Terms]) OR Asthma[MeSH Terms]) OR Prevalence[MeSH Terms]) OR Risk Factors[MeSH Terms]) OR Asthma/epidemiology [MeSH Terms] 2. Asthma epidemiology[Text Word]) OR Asthm*[Text Word]) OR Prevalent *[Text Word]) OR Sensitise *[Text Word]) OR Environ *[Text Word]) OR Exposure *[Text Word]) OR Symptom *[Text Word]) OR Respirator *[Text Word]) OR Allerg *[Text Word] 3. 6 OR 7 | Search sets 6-8 are the **MeSH terms** and **text words** for the  **concept** (asthma epidemiological studies). They are combined using the Boolean term **OR** |
| **Context(C)** | 1. Africa[MeSH Terms] 2. Africa *[Text Word]) OR Zambia[Text Word]) OR Swaziland[Text Word]) OR Sudan[Text Word]) OR Senegal[Text Word]) OR Angola[Text Word]) OR Gabon[Text Word]) OR Botswana[Text Word]) OR Benin[Text Word]) OR Rwanda[Text Word]) OR Malawi[Text Word]) OR Cameroon[Text Word]) OR Gambia[Text Word]) OR Congo[Text Word]) OR Mozambique[Text Word]) OR Tanzania[Text Word]) OR Algeria[Text Word]) OR Zimbabwe[Text Word]) OR Ghana[Text Word]) OR Kenya[Text Word]) OR Uganda[Text Word]) OR Egypt[Text Word]) OR Ethiopia[Text Word]) OR Niger[Text Word]) OR Nigeria[Text Word]) OR South Africa[Text Word]) OR Saharan[Text Word] 3. 9 OR 10 | Search sets 9-11 are the **MeSH terms** and **text** **words** for the  **context** (Africa/African countries). They are combined using the Boolean term **OR** |
| **Combination of P,C & C** | 1. 5 and 8 and 11 2. Asthm*[Title/Abstract] 3. 12 and 13 4. 14 and date filter (1990/01/01 to 2019/08/19) 5. 15 and species filter (humans) 6. 16 and language filter (English) 7. 17 and article type filter (Observational study) 8. 18 and article type filter (Multi-centre study) 9. 19 and article type filter (Journal article) 10. 20 and article type filter (Comparative study) 11. 21 NOT “African-American” | Search sets 12-22 combine the PCC elements using the Boolean term **AND**. Search filters for date, language, and article type were also included. |
