# Supplementary material for: Assessing the nature of asthma in African epidemiological studies: a scoping review protocol
Source: Syst Rev. 2020 Oct 7;9:230. doi: 10.1186/s13643-020-01491-7 (PMC7539529; doi:10.1186/s13643-020-01491-7)
Supplement: Supplementary file 3 — Additional file 3. Data charting form. [file 13643_2020_1491_MOESM3_ESM.docx]

**Additional File 3: Data charting form**

|  | **Reporting status: Reported (✓) or Not reported (X)** | **Description** |
| --- | --- | --- |
| **Study Details** | |  |
| Author(s) |  |  |
| Year of publication |  |  |
| Country |  |  |
| Study setting |  |  |
| **Inclusion/Exclusion Criteria** | |  |
| Study design |  |  |
| Population characteristics |  |  |
| Outcome |  |  |
| **Study Details and Characteristics** | |  |
| Sample size |  |  |
| Sampling |  |  |
| Data collection methods (tools and techniques) for variable measurement |  |  |
| Participants (details e.g. age/sex and number) |  |  |
| Definition of asthma phenotype |  |  |
| Identified risk factors(lifestyle and environmental, presence of comorbidities) |  |  |
| Identified confounders |  |  |
| **Details/Results extracted from study** (in relation to the concept of the scoping review) | |  |
| Key results based on review objectives |  |  |
| Clarity of interpretation of results |  |  |
| Discussion of limitations |  |  |
| Recommendations for future research given |  |  |
| Assessment of generalizability of findings |  |  |
